# Novel UDP-GalNAc Derivative Structures Provide Insight into the Donor Specificity of Human Blood Group Glycosyltransferase*

**DOI:** 10.1074/jbc.M115.681262

**Published:** 2015-11-02

**Authors:** Gerd K. Wagner, Thomas Pesnot, Monica M. Palcic, Rene Jørgensen

**Affiliations:** From the ‡ Department of Chemistry, King's College London, Faculty of Natural & Mathematical Sciences, Britannia House, 7 Trinity Street, London SE1 1DB, United Kingdom,; the § University of East Anglia, School of Pharmacy, Norwich NR47TJ, England, and; the ¶ Carlsberg Laboratory, Gamle Carlsberg Vej 10, DK-1799, Copenhagen V, Denmark

**Keywords:** crystallography, glycosyltransferase, human, kinetics, nuclear magnetic resonance (NMR), UDP-GalNAc derivative, donor specificity

## Abstract

Two closely related glycosyltransferases are responsible for the final step of the biosynthesis of ABO(H) human blood group A and B antigens. The two enzymes differ by only four amino acid residues, which determine whether the enzymes transfer GalNAc from UDP-GalNAc or Gal from UDP-Gal to the H-antigen acceptor. The enzymes belong to the class of GT-A folded enzymes, grouped as GT6 in the CAZy database, and are characterized by a single domain with a metal dependent retaining reaction mechanism. However, the exact role of the four amino acid residues in the specificity of the enzymes is still unresolved. In this study, we report the first structural information of a dual specificity cis-AB blood group glycosyltransferase in complex with a synthetic UDP-GalNAc derivative. Interestingly, the GalNAc moiety adopts an unusual yet catalytically productive conformation in the binding pocket, which is different from the “tucked under” conformation previously observed for the UDP-Gal donor. In addition, we show that this UDP-GalNAc derivative in complex with the H-antigen acceptor provokes the same unusual binding pocket closure as seen for the corresponding UDP-Gal derivative. Despite this, the two derivatives show vastly different kinetic properties. Our results provide a important structural insight into the donor substrate specificity and utilization in blood group biosynthesis, which can very likely be exploited for the development of new glycosyltransferase inhibitors and probes.

## Introduction

**FIGURE 1.**
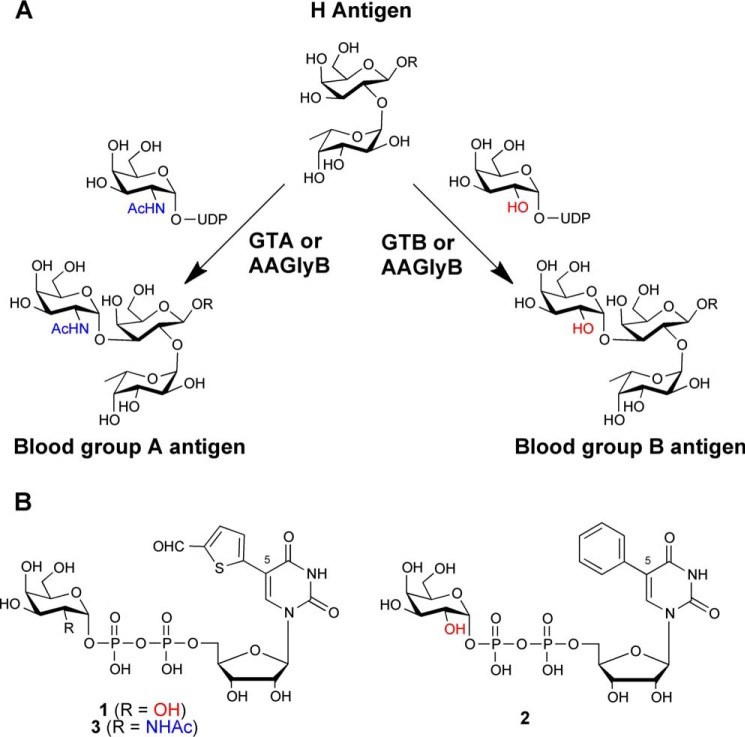
**The ABO(H) antigen synthesis and structures of UDP-sugar analogues.** *A*, reactions catalyzed by blood group glycosyltransferases GTA, GTB, and AAGlyB. *B*, structures of UDP-sugar analogues used in this study or discussed in the text. **1** is (5-(5-formylthien-2-yl)-UDP-α-d-galactose), **2** is (5-(phenyl)-UDP-α-d-galactose), and **3** is (5-(5-formylthien-2-yl)UDP-α-d-galactosamine).

Glycosyltransferases (GTs)^3^ are vital enzymes responsible for the synthesis of oligosaccharides, polysaccharides, and glycoconjugates (1, 2). One important example is the generation of human ABO(H) blood group A and B antigens. These antigens differ in only a single sugar (A: *N*-acetyl d-galactosamine; B: d-galactose), which is installed at the common H-antigen core by one of two closely related GTs, an α-(1→3)-*N*-acetylgalactosaminyltransferase (GTA) and an α-(1→3)-galactosyltransferase (GTB) (Fig. 1*A*). Despite the different donor substrate specificities GTA and GTB are identical except for only four amino acids. GTA has residues Arg^176^, Gly^235^, Leu^266^, and Gly^268^, whereas GTB has residues Gly^176^, Ser^235^, Met^266^, and Ala^268^ at the corresponding positions (3–5). The structural basis for this difference in specificity between GTA and GTB is currently unresolved. To address this question, important insights can be gleaned from structural and mechanistic studies with mutant blood group enzymes. One naturally occurring blood group GT mutant is the cis-AB enzyme, AAGlyB, which can bind and utilize either UDP-Gal or UDP-GalNAc with almost equal efficiency (3, 6). In AAGlyB the four residues critical for donor recognition are Arg^176^, Gly^235^, Gly^266^, and Ala^268^ with Gly at the third position belonging to neither A nor B.

Small molecular inhibitors and substrate analogues are useful tools to investigate the details of the substrate recognition and mechanism in GTs (7–10). Recently, we have used the human blood group enzymes as a model system for the development of a new class of GT donor analogues derived from the natural UDP-Gal donor substrate of galactosyltransferases (7, 11–14). These donor analogues carry an additional substituent in position 5 of the uracil base (Fig. 1*B*). Intriguingly, this modification in position 5 of UDP-Gal turned the donor analogues **1** (5-(5-formylthien-2-yl)-UDP-α-d-galactose) and **2** (5-(phenyl)-UDP-α-d-galactose) into potent inhibitors with very low turnover rates with the AAGlyB mutant (7, 12). Previous studies of **1** and **2** using the AAGlyB cis-AB mutant have provided important insights into the basis for this enzyme inhibition (7, 12). Structures of AAGlyB in complex with **1** and **2** show that the donor derivatives are binding in the active site with the galactose in the “tucked under” conformation as seen previously with native UDP-Gal (15–18).

However, the 5-formylthienyl and phenyl substituents in these compounds are obstructing the stacking of the side chain of Trp^181^ positioned in a flexible active site loop with the side chain of Arg^352^ in the equally flexible C terminus. Upon binding of native donor substrates to blood group GTs, this stacking interaction stabilizes the “closed” conformation and contributes to the H-antigen acceptor (HAA) binding site, which is required for full catalytic activity. Hence, obstruction of this crucial conformational transition provides an explanation for the inhibitory activity of **1** and **2** toward AAGlyB (7, 19). The structures of AAGlyB in complex with compounds **1** or **2** and the HAA substrate acceptor, α-l-Fuc*p*-(1→2)-β-d-Gal*p*, in conjunction with detailed enzymological data, also provided an explanation for the different binding affinities and turnover rates of the two different UDP-Gal derivatives (12). Intriguingly, the ternary structure of the AAGlyB-**1**-HAA complex revealed an alternatively closed conformation of the C terminus forming hydrogen bonds with the 5-formylthienyl substituent. This alternatively closed conformation was not present in the AAGlyB-**2**-HAA structure presumably because of the lack of hydrogen bonding opportunities between the C terminus and the 5-phenyl substituent in **2**. Therefore, the ability of AAGlyB to adopt this alternative conformation in **1** but not in **2** provided an explanation for the difference in residual rate of product formation between the two derivatives. It also suggested a reason for the elevated *K_m_* for acceptor substrate with compound **1** because a histidine residue in this alternative conformation of the C terminus is no longer hydrogen bonding to the acceptor.

Although several structures of human blood group GTs in complex with UDP-Gal have been reported (7, 12, 15), attempts to solve a structure with UDP-GalNAc have remained elusive due to the very rapid hydrolysis of the donor when soaked into the crystals. Therefore, we have expanded our ligand design to the UDP-GalNAc congener of inhibitor **1** (compound **3**, Fig. 1*B*) assuming that this UDP-GalNAc derivative would also behave as an inhibitor with a low turnover rate.

Herein, we present the synthesis of compound **3** as well as structures of AAGlyB in complex with **3** alone, and in complex with **3** together with the HAA-acceptor. Importantly, the structure of the AAGlyB-**3** complex contains the donor analogue with a partially intact GalNAc portion, which, for the first time, gives an indication of the binding mode of UDP-GalNAc to a human blood group GT. In this new structure, the GalNAc moiety is placed in the active site in a conformation, which is different from the canonical tucked under conformation previously observed in other UDP-sugar-GT complex structures (7, 12, 15–18, 20–24).

Importantly, this new GalNAc conformation is catalytically productive and, supported by enzyme kinetics, we suggest that donor hydrolysis along with additional conformational changes is a prerequisite for proper HAA binding. The new structures allow a direct comparison, for the first time, of the recognition and utilization of the two alternative donors UDP-Gal and UDP-GalNAc by AAGlyB, and offer a structural explanation for the dual specificity of this enzyme.

## Experimental Procedures

### 

#### Synthesis of UDP-GalNAc Derivative

##### **3**, Preparative Chromatography

Ion-pair chromatography was performed using Lichroprep RP-18 resin gradient 0–10% acetonitrile (or methanol) against 0.05 m triethylammonium bicarbonate over 480 ml at a flow rate of 5 ml/min. Product-containing fractions were combined and reduced to dryness. The residue was co-evaporated repeatedly with methanol to remove residual triethylammonium bicarbonate. Anion-exchange chromatography was performed using a MacroPrep 25Q resin, gradient 0–100% 1 m triethylammonium bicarbonate (pH 7.3) against H_2_O over 480 ml at a flow rate of 5 ml/min. Product-containing fractions were combined and reduced to dryness. The residue was co-evaporated repeatedly with methanol to remove residual triethylammonium bicarbonate. 5-Iodouridine-5α-monophosphate **5** (292 mg, 0.65 mmol) (19) was dissolved in dry dimethyl sulfoxide and co-evaporated (3 times) with dry *N*,*N*-dimethylformamide to remove residual water. The residue was dissolved in 0.5 ml of dry dimethyl sulfoxide. Morpholine (400 μl, 4.6 mmol) was added, and the mixture was stirred at room temperature for 5 min. Dipyridyl disulfide (500 mg, 2.3 mmol) and triphenylphosphine (600 mg, 2.3 mmol) were added at 5-min intervals. The reaction mixture was further stirred for 60 min at room temperature before being quenched with 0.1 m NaI in acetone until a colorless solid precipitated out of the solution. The supernatant was removed and the phosphoromorpholidate of **5** (sodium salt) was isolated by filtration as a colorless powder (354 mg, 99% yield). This material was sufficiently pure for analytical characterization and was used in the next step without further purification. δ*_H_* (400 MHz, D_2_O) 3.04–3.16 (4H, m, morph), 3.63–3.73 (4H; m, morph), 3.98–4.15 (2H, m, H-5′), 4.24–4.26 (1H, m, H-4′), 4.27–4.32 (1H, m, H-3′), 4.37 (1H, t, *J*_H,H_ 5.3 Hz, H-2′), 5.94 (1H, d, *J*_H,H_ 5.3 Hz, H-1′), 8.18 (1H, s, H-6); δ*_C_* (75.5 MHz, D_2_O) 44.2 (morph), 63.4 (d, *J*_C,P_ 5.3 Hz, C-5′), 66.3 (morph), 68.0 (C-5), 68.6 (C-3′), 73.2 (C-2′), 83.0 (d, *J*_C,P_ 8.5 Hz, C-4′), 88.2 (C-1′), 145.0 (C-6), 153.0 (C-2), 161.9 (C-4); δ_P_ (121.5 MHz, D_2_O) 11.0. *m/*z (ESI) 519.9968 [M + H]^+^, C_13_H_19_IN_3_O_9_P requires 519.9976.

##### 5-Iodo-UDP-GalNAc (**4**)

5-Iodouridine-5α-monophosphoromorpholidate (33 mg, 0.06 mmol, 1 eq) was co-evaporated with dry pyridine (3 × 5 ml). *N*-Acetylgalactosamine-1-phosphate in its tributylammonium salt form (ref or prep) (2.5 eq) was added to the dry morpholidate mixture and further co-evaporated (3 × 5 ml) with pyridine. The dry residue was dissolved in dry *N*,*N*-dimethylformamide (2 ml) under nitrogen atmosphere and tetrazole in dry acetonitrile (5 eq) was added. After 5 h, the reaction reached completion and all solvents were removed under reduced pressure. The crude residue was purified sequentially by ion-exchange and ion-pair chromatography to give the title compound in its triethylammonium salt form (1.6 eq.) as a colorless, glassy solid in 27% yield (15.7 mg). δ_H_ (400 MHz, D_2_O) 2.08 (3H, s, CH_3_), 3.70–3.76 (2H, m, H-6″), 3.96 (1H, dd, *J*_H,H_ 2.7 and 11.0 Hz, H-3″), 4.03 (1H, d, *J*_H,H_ 2.4 Hz, H-4″), 4.15–4.30 (5H, m, H-2″, H-5″, H-5′, H-4′), 4.33–4.39 (2H, m, H-2′, H-3′), 5.54 (1H, m, H-1″), 5.92 (1H, d, *J*_H,H_ 4.9 Hz, H-1′), 8.26 (1H, s, H-6); δ_C_ (75.5 MHz, D_2_O) 22.8, 59.3, 61.7, 65.8, 68.4, 69.1, 69.2, 70.3, 72.7, 74.4, 84.0, 89.5, 95.4, 146.7, 152.4, 163.9, 193.5; δ_P_ (121.5 MHz, D_2_O) -13.0 (d, *J*_P,P_ 21.2 Hz), −11.5 (d, *J*_P,P_ 21.2 Hz). *m*/*z* (ESI) 365.4814 [M-2H]^2−^, C_17_H_24_IN_3_O_17_P_2_ requires 365.4814.

##### 5-(5-Formylthien-2-yl) UDP-GalNAc (**3**)

A 2-necked round bottom flask with 5-iodouridine-5′-diphosphate-α-d-*N*-acetylgalactosamine **4** (12.0 mg, 16.4 μmol, 1 eq), Cs_2_CO_3_ (2 eq), and 5-formyl-2-thiopheneboronic acid (1.5 eq) was purged with N_2_. Triphenylphosphine-3,3′,3″-trisulfonic acid trisodium salt (0.0625 eq), Na_2_Cl_4_Pd (0.025 eq), and degassed H_2_O (4 ml) were added and the reaction was stirred under N_2_ for 1 h at 50 °C. Upon completion, the reaction was cooled to room temperature and the pH was adjusted to 7 with 1% HCl. The black suspension was filtered through a membrane filter (0.45 μm). The filter was washed with H_2_O and the combined filtrates were evaporated under reduced pressure. The residue was sequentially purified by ion exchange and ion-pair chromatography to give the title compound in its triethylammonium salt form (1.5 eq) as a glassy solid in 76% yield (11.9 mg). δ_H_ (400 MHz, D_2_O) 2.08 (3H, s, CH_3_), 3.70–3.77 (2H, m, H-6″), 3.88 (1H, dd, *J*_H,H_ 3.0 and 10.9 Hz, H-3″), 3.96 (1H, d, *J*_H,H_ 2.9 Hz, H-4″), 4.10–4.14 (1H, m, H-5″), 4.20–4.24 (1H, m, H-2″), 4.26–4.38 (3H, m, H-4′, H-5′), 4.40–4.49 (2H, 2t, *J*_H,H_ 4.9 and 4.9 Hz, H-2′, H-3′), 5.53 (1H, m, H-1″), 6.01 (1H, d, *J*_H,H_ 4.8 Hz, H-1′), 7.72 (1H, d, *J*_H,H_ 3.8 Hz, thienyl), 7.98 (1H, d, *J*_H,H_ 4.1 Hz, thienyl), 8.44 (1H, s, H-6), 9.77 (1H, s, CHO); δ_C_ (75.5 MHz, D_2_O) 22.8, 59.3, 61.7, 65.8, 68.4, 69.1, 70.3, 72.8, 74.4, 84.3, 90.0, 95.4, 109.6, 126.1, 139.2, 140.3, 142.2, 145.0, 151.3, 163.7, 184.8, 188.0; δ_P_ (121.5 MHz, D_2_O) −13.0 (d, *J*_P,P_ 21.2 Hz), −11.5 (d, *J*_P,P_ 21.2 Hz). *m*/*z* (ESI) 716.0555 [M − H]^−^, C_22_H_28_N_3_O_18_P_2_S requires 716.0569.

### Cloning, Purification, and Crystallization

The AAGlyB was cloned, expressed, and purified in *Escherichia coli* as previously described (12, 25, 26) and the crystallization condition was described in Refs. 7 and 12. The AAGlyB-**3** complex crystals were flash cooled in liquid N_2_ after adding a cryosolution consisting of reservoir solution including 20% glycerol and 25 mm compound **3** to a crystal drop and letting it soak for 30 min at 4 °C. The AAGlyB-**3**-HAA crystals were flash cooled in liquid N_2_ as for AAGlyB-**3** crystals but with an additional 25 mm α-Fuc*p*-(1→2)-β-Gal*p*-O-(CH_2_)_7_-CH_3_ (HAA) acceptor added to the cryosolution.

### Enzyme Kinetics

The *K_m_*, *k*_cat_, and *K_i_* values for UDP-Gal, UDP-GalNAc, **1**, and HAA acceptor with AAGlyB were previously described (7). To determine the *K_m_* and *k*_cat_ values for **3** we used capillary electrophoresis with tetramethylrhodamine (TMR)-labeled α-Fuc*p*-(1→2)-β-Gal*p*-O-(CH_2_)_8_CONH(CH_2_)_2_NH-CO acceptor (27) as described in detail for compound **2** in Ref. 12. For donor kinetics, **3** (25–0.29 μm), AAGlyB protein (0.004 μg/ml), TMR-labeled acceptor (25 μm), MnCl_2_ (20 mm), BSA (1 mg/ml), and MOPS buffer (50 mm, pH 7.0) were incubated at 37 °C. All concentrations are final concentrations in a volume of 12 μl. To avoid substrate depletion, the assay was only incubated for 8 min. For acceptor kinetics with **3**, the following concentrations were used: **3** (33.3 μm) and TMR-labeled acceptor (250–1.9 μm), incubated for 20 min. The *K_m_* and *V*_max_ values were obtained by fitting the data points to a Michaelis-Menten curve using GraphPad Prism 4 (GraphPad Software). The *K_i_* value for **3** with AAGlyB competing with UDP-[6-^3^H]Gal transfer was determined by a standard SepPak reverse-phase radiochemical assay as previously described (15). *K_i_* values were determined by linear regression of Dixon plots using 100 μm HAA, 2 μm UDP-Gal, 300,000 dpm of UDP-[6-^3^H]Gal, and 0, 0.5, 1, and 2 μm **3**. Because the GalNAc moiety in compound **3** is not radiolabeled this assay set-up does not differentiate between inhibitor and substrate behavior of **3**. Reduced incorporation of labeled [6-^3^H]Gal from UDP-[6-^3^H]Gal can be the result either of inhibition, or incorporation of unlabeled GalNAc. Therefore, the determination of *K_i_* values in this assay is compatible with the observed donor substrate behavior of **3**.

### Dowex Hydrolysis Assay

Hydrolysis of UDP-Gal and UDP-GalNAc by AAGlyB was followed with a radiochemical assay, as previously described (28) using the following conditions: 50 mm MOPS (pH 7), 20 mm MgCl_2_, 1 mg/ml of bovine serum albumin, 300 μm UDP-Gal or UDP-GalNAc, 300,000 dpm of UDP-[6-^3^H]Gal or UDP-[6-^3^H]GalNAc, 350 and 35 μm enzyme for the UDP-Gal and UDP-GalNAc assays, respectively. The 10-μl reaction mixtures were incubated in a thermo block at 37 °C for 60 min. The reaction was terminated by adding 300 μl of ice-cold H_2_O and transferred to Mini-columns (5 ml, Sigma), which were packed with 0.4 g of AG1-X8 Resin (chloride 200–400 mesh, Bio-Rad). The reaction product was washed first with 400 μl and then 500 μl of H_2_O by brief centrifugation and transferred to a scintillation vial. Ten milliliters of Ecolite^TM^ + scintillation fluid (MP Biomedicals) was added, and radioactivity was measured in a LS 6500 scintillation counter (Beckman Coulter^TM^).

### NMR Experiments

NMR was used to study the stereochemistry of the hydrolysis reaction. In these experiments the reaction medium consisted of 500 μm UDP-GalNAc and 17.5 μm AAGlyB or 250 μm **3** and 30 μm AAGlyB, in 50 mm deuterated BisTris buffer (pH 7) containing 10 mm MgCl_2_. After mixing donor and enzyme the NMR spectra were recorded at 291 K on a Bruker Advance 800 instrument at 799.96 MHz for proton using a 5-mm cryo probe. The assignment was based on Bruker standard experiments as two-dimensional DQFCOSY, NOESY, and TOCSY recorded with 4 K data points and 512 increments and an acquisition time of 0.23 s, 0.6-s NOESY mixing time, and 0.08-s spinlock in the TOCSY experiment.

### Data Collection, Reduction, and Structure Solution

X-ray diffraction data were collected to 1.9-Å resolution on the AAGlyB-**3** complex at beamline I911-2 at the MAX II synchrotron in Lund, Sweden (λ = 1.038 Å, 100 K). X-ray diffraction data on the AAGlyB-**3**-HAA complex was collected to 1.65-Å resolution at beamline BL14.1 at the BESSY II synchrotron in Berlin, Germany (λ = 0.918 Å, 100 K). Both data sets were integrated and scaled by XDS (29). Subsequently, the structures were solved by molecular replacement using the module Phaser (30) including the solved structure of wild-type GTB (PDB entry 2RIT) as a search model. The two structures belong to space group P2_1_2_1_2 with two molecules in the asymmetric unit. Restrained refinement was initially carried out in REFMAC5 (31) before iteratively rebuilding the structures using Coot (32) and finally refining in Phenix (33) with TLS included. Ramachandran plots were calculated in PROCHECK (34) and show that all residues are within the allowed region in both structures. The crystallographic details are presented in Table 1. The structural figures were all prepared in PyMOL (DeLano Scientific LLG).

**TABLE 1:**
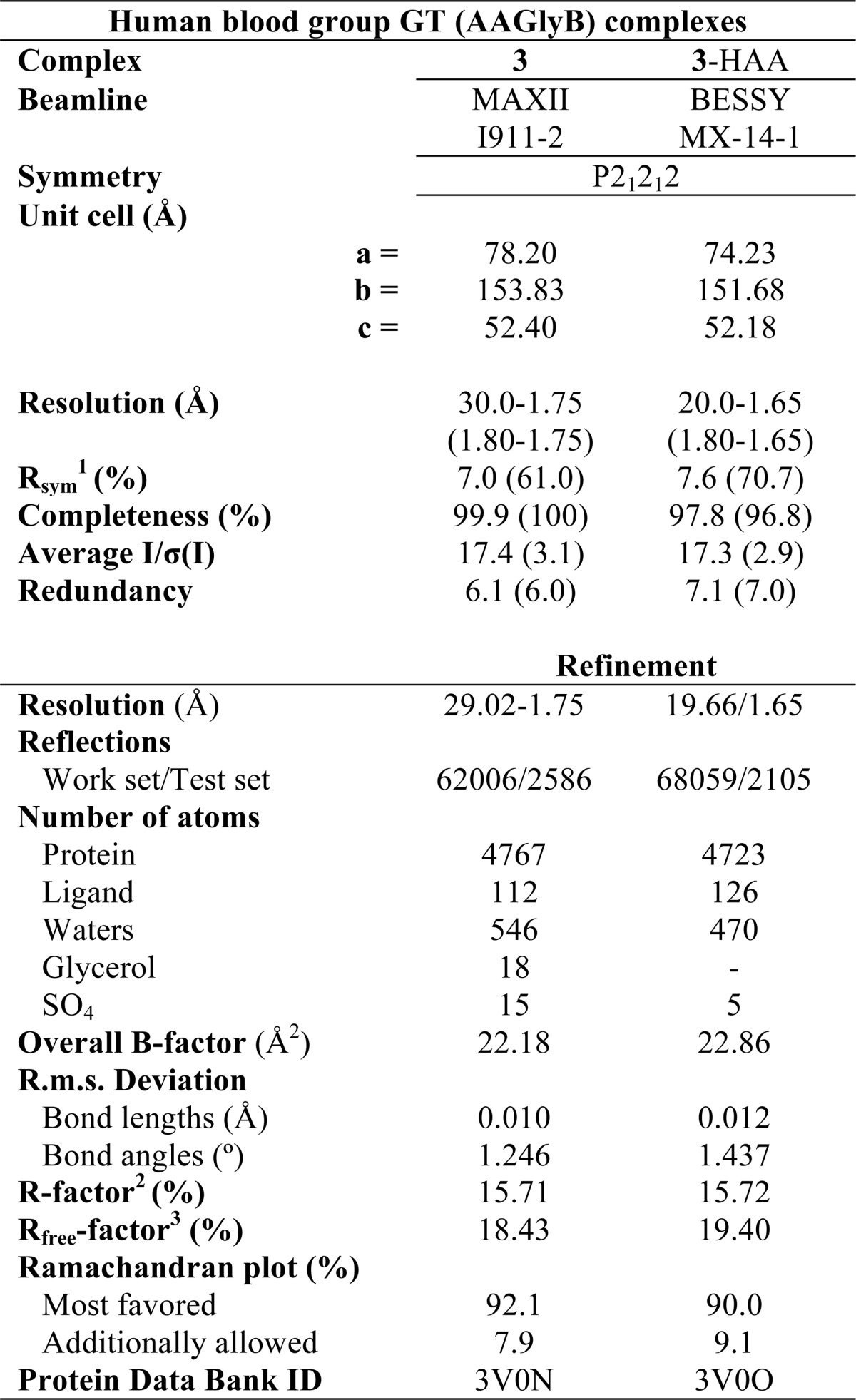
**Statistics for data collection and refinement**

*^a^* Values in parenthesis are for the highest resolution shell.

*^b^ R*_sym_ = Σ|(*I* − <*I*>)|*I*Σ(*I*), where *I* is the observed intensity.

*^c^ R* = Σ‖*F*_obs_| − |*F*_calc_‖Σ|*F*_obs_|, where |*F*_obs_| and |*F*_calc_| are observed and calculated structure factor amplitude.

*^d^* The *R*_free_ value was calculated with a random 5% subset of all reflections excluded from refinement.

## Results

### 

#### 

##### Synthesis

**SCHEME 1.**
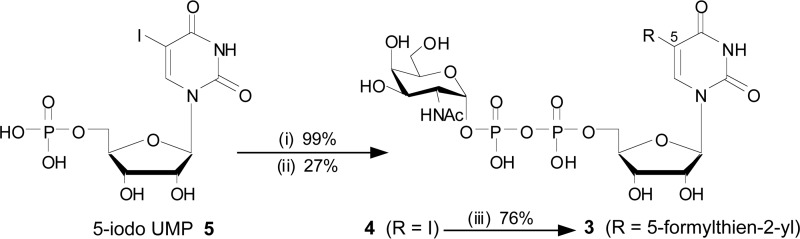
**Synthesis of UDP-GalNAc derivative 3**. Reagents and conditions: (i) morpholine, 2,2′-dipyridyldisulfide, PPh_3_, dimethyl sulfoxide, room temperature, 1 h; (ii) α-d-GalNAc-1-phosphate, tetrazole, MeCN, *N*,*N*-dimethylformamide, room temperature, 5 h; (iii) 5-formyl-2-thiopheneboronic acid, Cs_2_CO_3_, triphenylphosphine-3,3′,3″-trisulfonic acid trisodium salt, Na_2_Cl_4_Pd, H_2_O, 50 °C, 1 h.

For the preparation of the new UDP-GalNAc derivative **3** we adapted our previously developed protocol for the Suzuki-Miyaura cross-coupling of unprotected sugar-nucleotides (Scheme 1) (7, 35–37). 5-Iodo-UDP-GalNAc **4** was prepared from GalNAc-1-phosphate and 5-iodo-UMP **5** via conversion of the mononucleotide into the corresponding phosphoromorpholidate, followed by tetrazole-catalyzed pyrophosphate bond formation (38). Pleasingly, the free acetamido group in **4** did not interfere with the cross-coupling reaction in the next and final synthetic step. Thus, a water-soluble catalyst was used to successfully cross-couple the complete sugar nucleotide **4** with 5-formyl-2-thiopheneboronic acid to give the target 76% yield of UDP-GalNAc derivative **3**.

##### Enzyme Kinetics

The kinetic parameters for the glycosyl transfer reaction of AAGlyB with UDP-Gal derivative **1** as donor and the HAA acceptor were described previously (Table 2) (7, 12). These results showed that the 5-formylthienyl substituent in **1** led to efficient inhibition of transferase activity. In this study, using a capillary electrophoresis assay with TMR-labeled acceptor as previously described (12) we determined the *K_m_* and *k*_cat_ values of the new UDP-GalNAc donor analogue, **3**, with AAGlyB. Interestingly, the enzymological profile of **3** is markedly different from that of **1** (Table 2). Donor analogue **3**, which differs from **1** only in the nature of the sugar, has a *K_m_* value similar to that of **1**, but is turned over about 25-fold faster, despite the identical nature of the 5-substituent in **1** and **3**. Overall, **3** has a very similar enzymological profile to its 5-unsubstituted parent UDP-GalNAc, with a *K_m_* that is about 3-fold lower and similar values for *k*_cat_ and *K_m_* for acceptor. This suggests that, although **3** binds to the enzyme with similar affinity as **1**, the 5-formylthienyl substituent is not sufficient in this case to prevent product formation. Interestingly, in conjunction with **3** behaving as a natural substrate the acceptor *K_m_* is also not elevated compared with the acceptor *K_m_* for the reaction using UDP-GalNAc. This is in contrast to the higher *K_m_* value for acceptor when using **1**, which has a much lower turnover rate and it may suggest that donor hydrolysis is a prerequisite for proper acceptor binding. Finally, we also evaluated the ability of **3** to compete with UDP-Gal binding to AAGlyB in a radiochemical assay. As previously seen for **1,** compound **3** competes for binding with a *K_i_* value, which is in the same range as *K_m_* for **3**. However, because compound **3** behaves similar to the natural UDP-GalNAc substrate the reduced incorporation of labeled [6-^3^H]Gal is most likely the result of incorporation of unlabeled GalNAc.

**TABLE 2:**
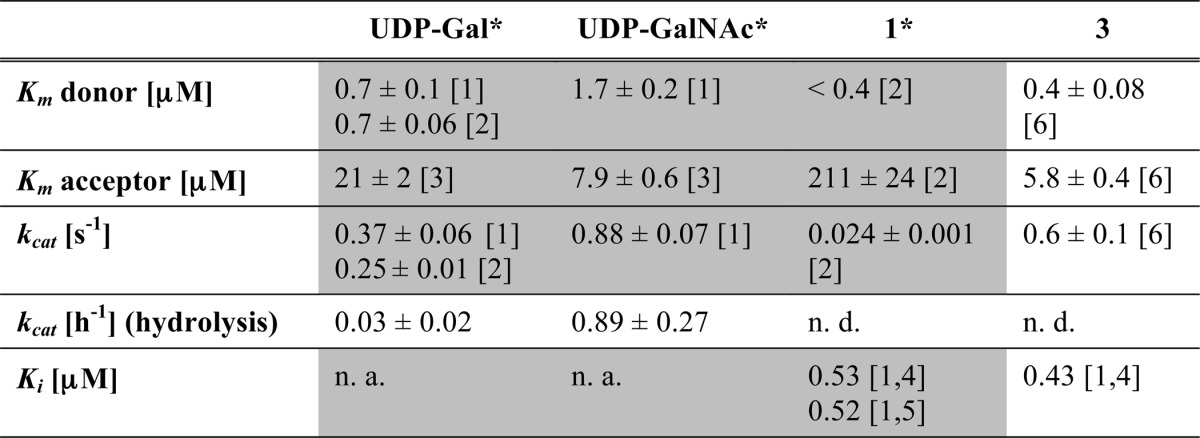
**Enzymological characterization of compound 3 with AAGlyB**

The grey shaded area indicates previously reported values. *Kinetic values were previously reported in Ref. 7. [1] Radiochemical assay, with 100 μm acceptor; [2] HPLC assay; [3] radiochemical assay, with 100 μm donor; [4] donor: UDP-Gal (2 μM); [5] donor: UDP-GalNAc (2 μM). [6] Capillary electrophoresis assay with TMR labeled HAA FucGal acceptor. n.a., not applicable. n.d., not determined.

##### Crystallographic Analysis

Although a structure of a human blood group GT in complex with a deoxy-antigen acceptor (DA) and intact UDP-Gal has previously been reported (15), a structure with the alternative UDP-GalNAc donor has remained elusive. AAGlyB catalyzes the hydrolysis of UDP-GalNAc with a rate that is ∼30 times higher than for UDP-Gal (Table 2). This is in keeping with previous findings with wild-type blood group enzymes, that the hydrolysis of UDP-GalNAc is catalyzed much more readily by GTA than the hydrolysis of UDP-Gal by GTB (39).

To further examine the enzymological profile of the UDP-GalNAc derivative, **3**, and in particular its unexpected donor substrate activity, we solved two crystal structures of AAGlyB in complex with **3**. One complex is with **3** alone and one complex is with the HAA acceptor in the binding site. The statistical details for the x-ray data collection and refinement of the AAGlyB structures are shown in Table 1. The data diffracted to a maximum resolution of 1.75 and 1.65 Å with final *R*_free_ values of 18.43 and 19.4% for AAGlyB-**3** and AAGlyB-**3**-HAA, respectively. Both structures belong to space group P2_1_2_1_2 containing 2 molecules in the asymmetric unit and with similar unit cell parameters. The two structures are highly isomorphous except for the last 10 residues of the C terminus, which are only visible in chain A of AAGlyB-**3**-HAA (residues 345–354).

##### AAGlyB Structure in Complex with **3**

**FIGURE 2.**
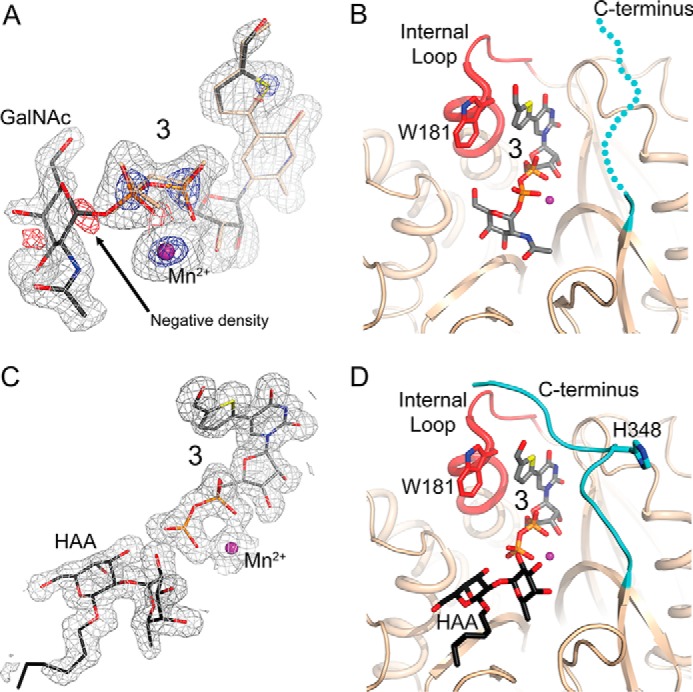
**Crystal structures of AAGlyB in complex with 3, with and without acceptor.** *A*, electron density of the donor binding site in AAGlyB-**3**. An anisotropically refined simulated annealing *F_o_* − *F_c_* omit map around **3**. *Gray* density is contoured at 2.5 σ, the *blue* density is the same map contoured at 12.0 σ emphasizing the strong peaks of the S, P, and Mn^2+^ atoms. The *red* density is a *F_o_* − *F_c_* map of the final refined structure including **3** (contoured at −4.0 σ). The negative density at the O1-C1 bond is marked by an *arrow. B*, the AAGlyB-**3** complex showing an ordered internal loop (*red*) and a disordered C terminus (*cyan*) as illustrated by a *dotted line*. Compound **3** including the GalNAc is shown in *gray* carbon atoms. *C*, composite 2*F_o_* − *F_c_* omit map of **3**, Mn^2+^ and HAA in the AAGlyB-**3**-HAA complex (contoured at 1.0 σ). *D*, the AAGlyB-**3**-HAA complex showing an ordered internal loop in the “closed” conformation and the ordered C terminus. Colors as in *A*. The acceptor is shown in *black* carbon atoms.

By carrying out the crystal soaks at 4 °C we ultimately obtained a structure of the AAGlyB-**3** complex including a visible GalNAc in chain B of the two chains in the asymmetric unit (Fig. 2*A*). A difference map of the AAGlyB structure also shows hints of a GalNAc in two different conformations in the active site of chain A, but the quality of the map in this area did not permit building a plausible model of the two GalNAc conformations. In the AAGlyB-**3** structure the internal loop (residues 173–188) in both chains is well defined both in the presence and absence of acceptor. However, in contrast to the previously solved structures of AAGlyB-**1** and AAGlyB-**2** (12), the loop in AAGlyB-**3** is in the closed conformation upon binding of the donor analogue alone (Fig. 2*B*).

##### AAGlyB Structure in Complex with **3** and HAA

Soaking crystals with both **3** and HAA resulted in a structure with donor and acceptor binding sites occupied by a 5-formylthienyl UDP and a HAA, respectively (Fig. 2*C*). Binding of **3** and HAA triggers the C terminus of chain A to fold over the active site in the same alternative conformation (Fig. 2*D*) as previously seen in the AAGlyB-**1**-HAA structure (12), in which the donor analogue also has the 5-formylthienyl substituent. Similar to AAGlyB-**1**-HAA the hydrogen bond between HAA and His^348^ in AAGlyB-**3**-HAA is disrupted and is pointing away from the active site. In fact, the two structures are almost identical, as the only distinguishing factor, the sugar moiety, is not seen in either of the two complexes. The similarity to the binding of **1** is supported by our kinetic results, which show comparable *K_m_* values for both **1** and **3** (Table 2). However, the similar alternative binding mode for **1** and **3** suggests that the unusual conformation of the C terminus cannot be the reason for the much higher *K_m_* value for acceptor binding in the AAGlyB-**1**-HAA complex.

##### The GalNAc Conformation

**FIGURE 3.**
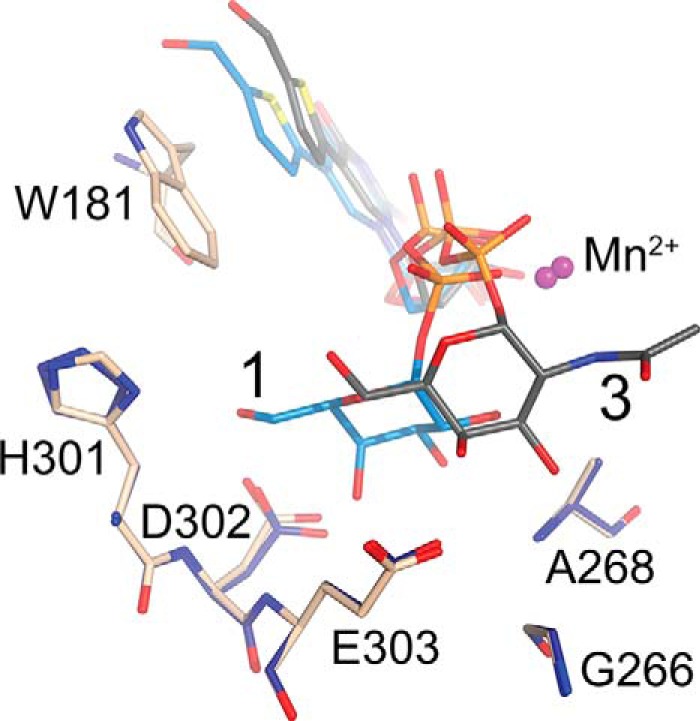
**Overlay of the AAGlyB-1 and AAGlyB-3 complex structures.** Compound **3** is shown in *gray* carbon sticks, **1** in *light blue* and surrounding residues in *light brown* for AAGlyB-**3** and *dark blue* for AAGlyB-**1**.

Interestingly, the AAGlyB-**3** structure shows the GalNAc ring in a conformation clearly different from the canonical tucked under conformation we have seen in previous blood group enzyme structures with UDP-Gal, **1,** or **2** and which is the conformation generally adopted by both UDP-Gal and UDP-GalNAc at a GT binding site (Fig. 3) (7, 12, 15–18, 20, 22–24). Additionally, this GalNAc conformation in AAGlyB is also different from the GalNAc conformation recently described in the similar, but metal-independent GT (CAZy group GT6) from *Bacteroides ovatus* (21).

**FIGURE 4.**
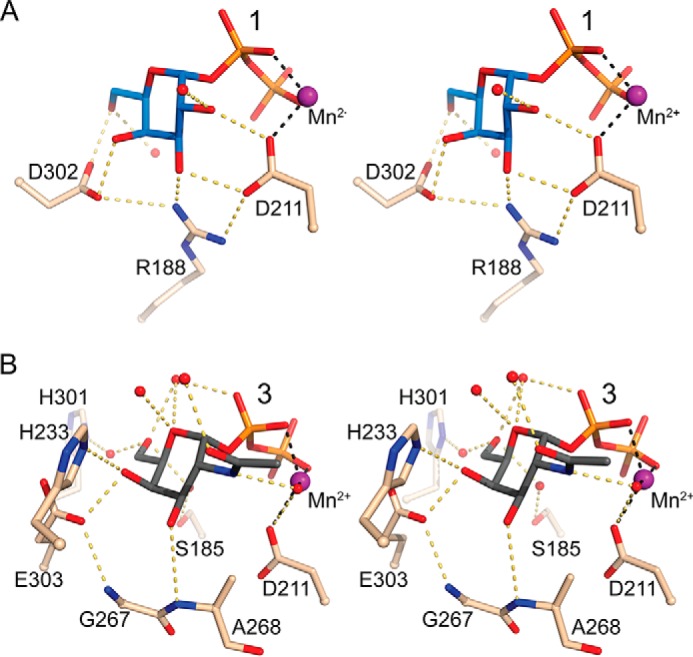
**Sugar recognition in 1 and 3 by AAGlyB.** *A*, stereo view of the AAGlyB-**1** binding site. H-bonds of the Gal moiety in **1** (*blue carbon sticks*) with nearby residues and waters (*red spheres*) are shown in *yellow dashes*. The coordination to Mn^2+^ is shown in *black dashes. B*, stereo view of the AAGlyB-**3** binding site. Compound **3** is shown in *gray* carbon sticks.

In the tucked under conformation in human blood group GTs, the hydroxyl groups of the Gal moiety are forming an elaborate network of hydrogen bonds to residues Arg^188^, Asp^211^, His^301^, and Asp^302^ (Fig. 4*A*, Table 3). In the AAGlyB-**3** structure the network of hydrogen bonds to the sugar is entirely different. Although O4′ of the GalNAc forms hydrogen bonds to Glu^303^ and His^233^, O3′ forms hydrogen bonds to the backbone of Gly^267^ and Ala^268^ (Fig. 4*B*, Table 3). Furthermore, in contrast to UDP-Gal in the tucked under conformation, several water molecules are bridging contact between the GalNAc in **3** and His^301^, Ser^185^, and Asp^211^.

**TABLE 3:** **Hydrogen bonds of donor analogue sugars**

Ligand atom	Interacting atoms			Distance	Ligand atom	Interacting atoms	Distance
Gal	AAGlyB-1	AAGlyB-2	2RJ7*^a^*		GalNAc	AAGlyB-3	
				Å			Å
O_2_	Asp^211^ Oδ2	Asp^211^ Oδ2	Asp^211^ Oδ2	2.8/2.5/3.1	N2	H_2_O^615^	2.6
O_2_	H_2_O^357^	H_2_O^1064^	-	2.9/3.1/ -	O_2_	H_2_O^741^	2.7
O3	Asp^211^ Oδ1	Asp^211^ Oδ1	Asp^211^ Oδ1	2.7/2.7/2.9	O3	Ala^268^ N	3.4
O3	Gly^267^ O	Gly^267^ O	Ala^268^ N	3.1/3.1/3.2	O4	Glu^303^ Oδ2	2.5
O3	Arg^188^ NH_2_	Arg^188^ NH_2_	Arg^188^ NH1	2.5/2.7/2.9	O4	His^233^ Nδ2	3.2
O4	Asp^302^ Oδ2	Asp^302^ Oδ2	Asp^302^ Oδ1	2.7/2.9/2.7	O4	Glycerol	2.4
O6	Asp^302^ Oδ1	Asp^302^ Oδ1	H_2_O^616^	2.6/2.8/2.8	O4	H_2_O^786^	3.2
O6	H_2_O^421^	Trp^181^ NE1	His^301^ Nδ1	2.8/2.7/2.9	O5	H_2_O^738^	3.1
					O5	H_2_O^786^	3.0
					O6	H_2_O^738^	2.9
					O6	H_2_O^650^	2.5
					O6	H_2_O^651^	2.8

*^a^* Hydrogen bond interactions of UDP-Gal in the human blood group GT complex, AABB-UDP-Gal-DA, (PDB entry 2RJ7) with UDP-Gal in the tucked under conformation.

**FIGURE 5.**
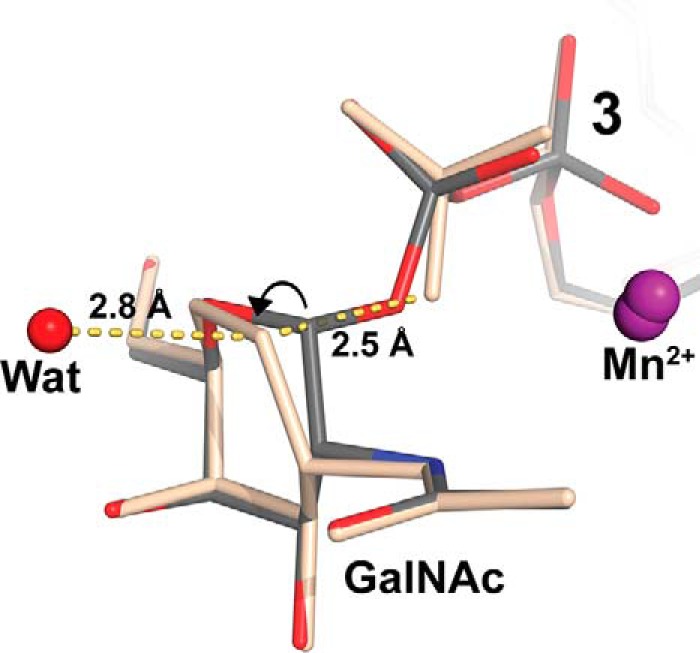
**UDP-GalNAc *versus* UDP + GalNAc in AAGlyB-3.** Result of refinement in Phenix with the GalNAc of **3** as a separate moiety (*light brown*) instead of **3** as an intact UDP-GalNAc donor. The distances from C1 to O3B of the β-phosphate and to the nearest water molecule, H_2_O^786^, are shown by *yellow dashes*.

The occupancy of the UDP-part of **3** in chain B is 100%, whereas the GalNAc moiety only has ∼60% occupancy. A simulated annealing *F_o_* − *F_c_* omit map of the active site of chain B shows a small gap between the GalNAc and the β-phosphate (Fig. 2*A*). In addition, a simulated annealing *F_o_* − *F_c_* map of the final refined structure with 60% occupancy of intact **3** and 40% of **3** without the GalNAc shows a small blob of negative density at the position of the glycosidic C1-O3B bond between the UDP β-phosphate and GalNAc. These electron density maps show that, for at least a fraction of the compound **3** molecules bound to enzyme in the crystal, the glycosidic bond is broken. Importantly, this implies that even in a “non-tucked under” conformation, the AAGlyB in the crystal can still hydrolyze the glycosidic bond of **3**. Also, a difference electron density map of the AAGlyB-**3** structure indicates that there are two slightly different positions of Asp^213^ in the D*X*D motif and the UDP phosphates (not shown). Furthermore, the strong electron density peak of the Mn^2+^ ion is also slightly elongated, indicating that there may be an alternative slightly shifted position of the metal. The two slightly different conformations could be a result of the cleavage of the glycosidic bond, which then releases the strain on the nucleotide and causes a shift of the α- and β-phosphates, the Mn^2+^ and Asp^213^. We chose to model an intact UDP-GalNAc donor analogue because this could be done without distortion of the stereochemistry around the glycosidic bond. However, assuming the bond is cleaved and the last refinement job is run again this time with a separate GalNAc moiety, the *R*_free_ only goes up by ∼0.1% and the negative density disappears from the C1-O3B bond. Furthermore, C1 is pulled away from the β-phosphate, which seems to make the pyranose ring adopt a flattened geometry (Fig. 5). Because the electron density map shows no sign of an O1 attached to C1 it is possible that the cleaved GalNAc exists as the C2 deprotonation product with a C1-C2 double bond, *i.e.* as a glycal. Although the quality of the electron density for the GalNAc does not justify modeling such an intermediate, this type of stabilized intermediate in the absence of acceptor has previously been suggested for the retaining GT-B-folded enzymes *E. coli* glycogen synthase (40) and *Arabidopsis thaliana* sucrose synthase-1 (41).

**FIGURE 6.**
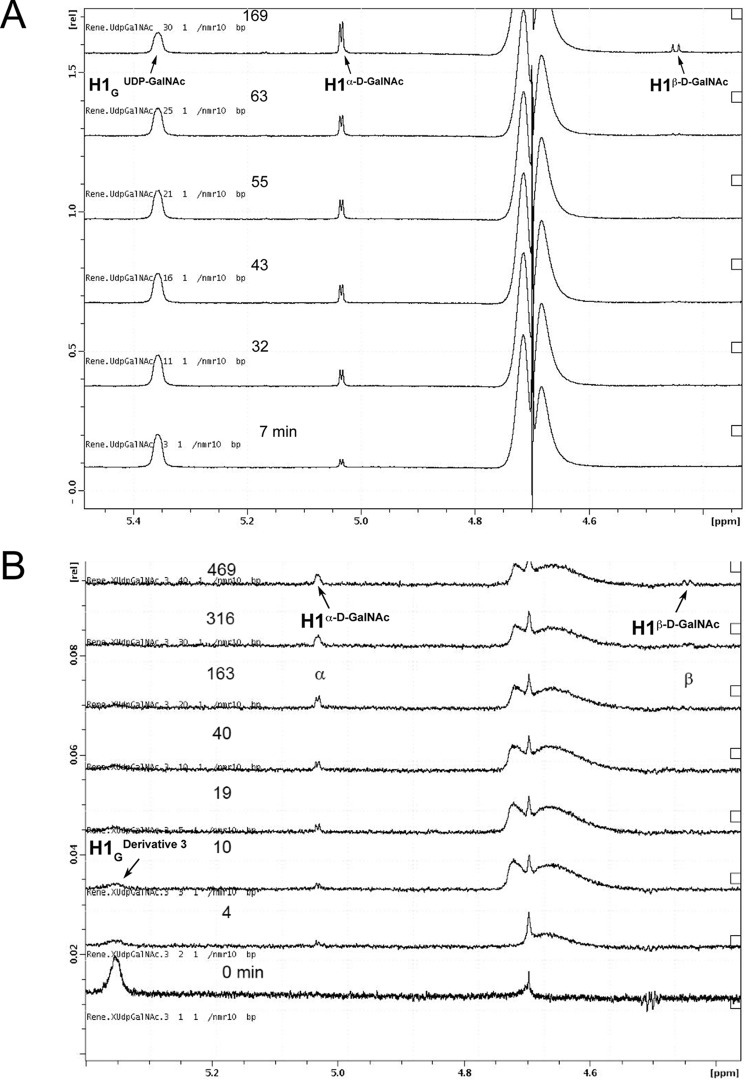
**^1^H NMR spectra of donor hydrolysis.** *A*, recorded during enzymatic hydrolysis of 500 μm UDP-GalNAc at 291 K in the presence of 17.5 μm AAGlyB. The spectra show that enzymatic hydrolysis releases α-*N*-acetylgalactosamine as a product that is subsequently transformed into β-*N*-acetylgalactosamine via mutarotation. *B*, recorded during enzymatic hydrolysis of 250 μm **3** at 291 K in the presence of 30 μm AAGlyB. The spectra show that enzymatic hydrolysis releases α-*N*-acetylgalactosamine as a product that is subsequently transformed into β-*N*-acetylgalactosamine via mutarotation. The reaction buffer in both experiments contained 50 mm BisTris-*d*_19_ (pH 7.0), 10 mm MgCl_2_.

Donor hydrolysis can be considered as transfer of the sugar to a water molecule, which is a poor acceptor substrate. In the refined position of the separate GalNAc there is a water molecule (H_2_O^786^) coordinated by the nearby glycerol, which seems to be in a perfect position to attack C1 from the opposite side of the UDP leaving group (Fig. 5). Such an attack would likely result in an inversion of configuration of the anomeric carbon during hydrolysis of the GalNAc in the absence of HAA acceptor. We followed the enzymatic hydrolysis of both the UDP-GalNAc donor substrate and compound **3** catalyzed by AAGlyB by ^1^H NMR (Fig. 6). The ^1^H NMR spectra show that enzymatic hydrolysis releases α-*N*-acetyl-d-galactosamine as a product that is subsequently transformed into β-*N*-acetyl-d-galactosamine via mutarotation. This is in accordance with previous experiments showing that hydrolysis of the UDP-Gal donor substrate by GTB also occurs with retention of configuration (28). However, these results indicate that this nearby H_2_O^786^ molecule seen in the AAGlyB-**3** structure does not attack the C1 atom from the opposite side of the leaving group.

**FIGURE 7.**
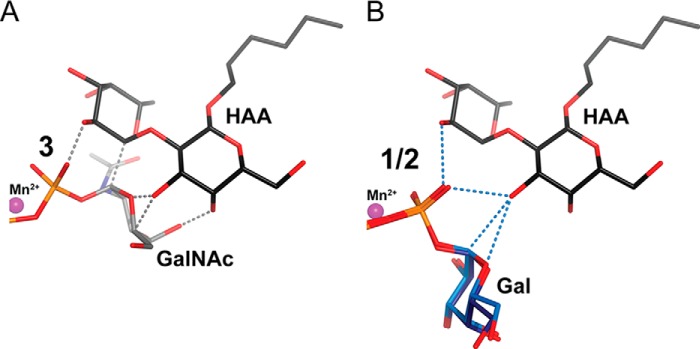
**Overlay of HA-acceptor onto intact donor derivatives.** *A*, the GalNAc part of AAGlyB-**3** is shown in *gray* carbon sticks, whereas the HAA part of AAGlyB-**3**-HAA is shown in *black* carbon sticks. *Gray dashed lines* indicate areas where the distance between compound **3** and HAA acceptor is 1.2–2.0 Å. *B*, the Gal part of AAGlyB-**1** and AAGlyB-**2** is shown in *dark* and *light blue* carbon sticks, respectively. HAA as in *panel A. Blue dashed lines* indicate areas where the HAA acceptor is between 2.7 and 3.0 Å from compounds **1** and **2**.

When comparing the binding site of the AAGlyB-**1**, AAGlyB-**2**, and AAGlyB-**3** and AAGlyB-**3**-HAA structures it can be seen that the HAA acceptor would not be able to bind to the active site without clashing significantly with the GalNAc seen in AAGlyB-**3** (Fig. 7*A*). Therefore, it is likely that the active site undergoes additional conformational changes to put the HAA and the GalNAc in a position suitably coordinated for reaction. In contrast, in the AAGlyB-**1** and -**2** structures with the galactose in the tucked under conformation the superimposed HAA appears to be able to fit simultaneously together with the donor (Fig. 7*B*). Here the HAA is potentially forming hydrogen bonds to O1 of the donor galactose as well as to a β-phosphate oxygen. Furthermore, O3 of the HAA galactose is ∼3 Å away from C1 of the donor galactose. However, because the anomeric carbon is retaining its conformation after transfer, here too, additional conformational changes are needed for obtaining a coordination suitable for the reaction.

## Discussion

**FIGURE 8.**
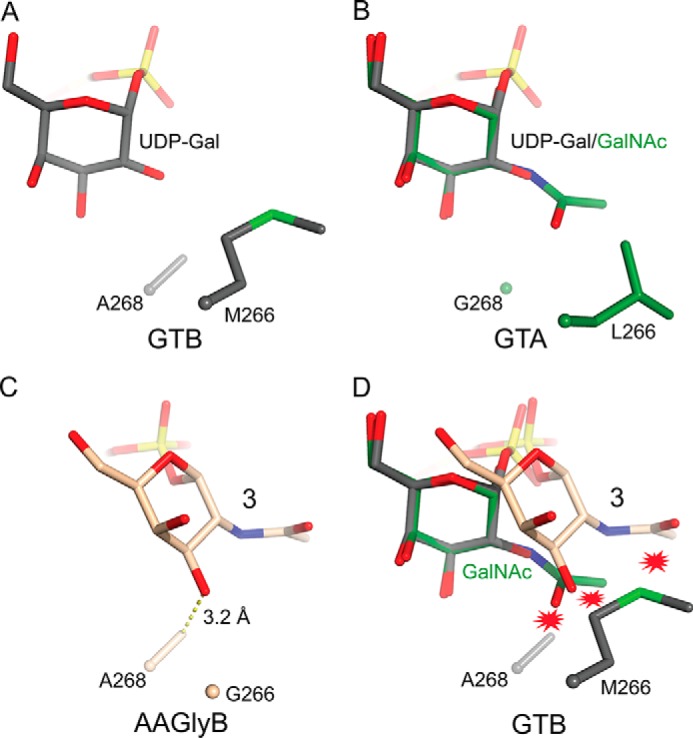
**Specificity model for human blood group GTs.** *A*, the GTB specific AABB-UDP-Gal-DA structure (Protein Data Bank entry 2RJ7) with the Gal in the tucked under conformation. *B*, the GTA structure (Protein Data Bank entry 1LZI) superimposed onto AABB-UDP-Gal-DA with a GalNAc modeled onto the Gal. This model shows that GTA residues Leu^266^ and Gly^268^ would readily accommodate a GalNAc in the tucked under conformation. *C*, the position of the GalNAc in the AAGlyB-**3** structure. The *dashed line* illustrates the distance between Ala^268^ and O3 of the GalNAc. *D*, superposition of the two GalNAc conformations in B and C onto AABB showing that both conformations of the GalNAc are clashing with the GTB residues Met^266^ and Ala^268^.

Our results provide new structural insights into the factors that govern substrate specificity in GTs. AAGlyB is unusual in that this GT can use two different UDP-sugars as donor, with near equal efficiency. The new results help refine our understanding of the structural basis for this dual specificity of AAGlyB. Previous kinetic studies using GTA/GTB chimeric enzymes have shown that residues 266 and 268 are the key residues that determine A/B substrate specificity (25, 42). In GTB, residues 266 and 268 are an alanine and methionine, respectively, which readily accommodate the Gal moiety in the tucked under conformation as illustrated in the previously solved GTB-specific AABB-UDP-Gal-DA structure (Fig. 8*A*). In GTA, Leu^266^ and Gly^268^ seem to leave sufficient space to accommodate the *N*-acetyl group of the UDP-GalNAc donor when superimposing a GalNAc moiety onto the Gal in the tucked under conformation (Fig. 8*B*). This is in contrast to both GTB and AAGlyB, both of which have an alanine residue in position 268. From the new AAGlyB-**3** structure it appears that Ala^268^ is at least partly responsible for preventing the GalNAc ring in **3** from adopting the tucked under conformation because the *N*-acetyl moiety of **3** would potentially be clashing with the short side chain of this alanine (Fig. 8*C*). When superimposing the GTB residues, Met^266^ and Ala^268^, together with the GalNAc from the AAGlyB-**3** structure and the GalNAc modeled in the tucked under conformation, the Met^266^ is clashing severely with both GalNAc conformations (Fig. 8*D*). In addition, when adding hydrogen atoms to the molecules using the validation program MolProbity (43) it is evident that not only Met^266^ but also Ala^268^ sterically overlaps with the *N*-acetyl group (results not shown). Hence, GTB residues at position 266 and 268 are preventing GalNAc from binding in any of the two conformations. In AAGlyB, residue 266 is a glycine, which allows, in conjunction with Ala^268^, the accommodation of the GalNAc residue in an alternative conformation. Although the presence of Ala^268^ therefore seems to preclude the GalNAc moiety of **3** from adopting the tucked under conformation in AAGlyB, it does permit, in conjunction with Gly^266^, the orientation of the donor in the alternative and productive conformation, despite the presence of the sterically demanding *N*-acetyl group.

Taken together, these results therefore provide an explanation for the dual specificity of the AAGlyB mutant, and may also offer an explanation for the different donor substrate specificities of the wild-type blood group GTs, GTA and GTB. Although we cannot rule out a tucked under conformation of the GalNAc in wild-type GTA, the AAGlyB-**3** structure provides valuable information on the function and importance of the two key residues, 266 and 268 in determining the specificity of the blood group GTs. Importantly, this mechanism of donor recognition and discrimination may also be used by other GTs. For example, the equivalent residues 266 and 268 in the Forssman synthetase, a close homologue of blood group synthases in GT6, are glycine and alanine as in AAGlyB (44). The Forssman synthetase is an α3-GalNAc transferase that transfers a GalNAc residue onto the glycolipid called the Forssman antigen. A hypothesis could be that, for the enzyme to allow the donor to adopt a productive configuration and act as an α3-GalNAc transferase, the active site of the enzyme must comprise a glycine residue either in position 266 (as in AAGlyB and FS) or 268 (as in GTA).

Finally, it is notable that the kinetic parameters for **3**, including *k*_cat_ and acceptor *K_m_*, are comparable with those of its parent molecule UDP-GalNAc (Table 2), despite the clashing position of the GalNAc with HAA. In contrast, the kinetic profile of **1** differs markedly from that of its parent UDP-Gal by having a lower *k*_cat_ and a higher acceptor *K_m_*. Furthermore, because both AAGlyB-**1**-HAA and AAGlyB-**3**-HAA are forming the alternative conformation of the C terminus the lack of hydrogen bonding between HAA and the C-terminal His^348^ residue cannot be the only factor contributing to the elevated acceptor *K_m_* in the case of **1**. Instead, it is more likely that acceptor binding is preceded by additional conformational changes and/or donor hydrolysis.

In summary, we show for the first time, the structure of a human blood group GT, AAGlyB, in complex with a UDP-GalNAc derivative. Intriguingly, this structure adopts a conformation different from the canonical tucked under conformation. The new donor conformation of the GalNAc in **3** is induced by the presence of residues, Gly^266^ and Ala^268^, important determinants for donor substrate specificity in blood group GTs. The structure of the AAGlyB-**3** complex therefore provides, for the first time, a detailed structural explanation for the dual specificity of AAGlyB. These insights may prove useful for ongoing efforts to expand the donor substrate specificity of GTs for synthetic applications. Finally, the new structural data shows the AAGlyB-**3**-HAA complex with a well defined internal loop and C terminus in the “pseudo-closed” conformation as also seen for the AAGlyB-**1**-HAA complex. This confirms that the nature of the substituent is important for the alternative closure of the binding pocket and will facilitate the design of new substituents with higher binding affinity of the donor.

## Author Contributions

R. J. collected the x-ray diffraction data, solved the AAGlyB-3 and AAGlyB-3-HAA crystals structures, as well as carried out the crystallographic analysis and the enzymological studies. G. K. W. designed the inhibitors and T. P. carried out the synthetic work. R. J. and M. M. P. designed the study and analyzed the data and discussed the results. R. J. wrote the paper and prepared the crystallographic figures and tables, with contributions from the other authors. All authors commented on the manuscript.

## Footnotes

3 The abbreviations used are:

GT: glycosyltransferase
GTA: human ABO(H) blood group α-(1→3)-*N*-acetylgalactosaminyltransferase
GTB: human ABO(H) blood group B α-(1→3)-galactosyltransferase
HAA: H antigen acceptor α-l-Fuc-(1→2)-β-d-Gal-O-R
DA: 3-deoxy-Gal-H antigen acceptor
TMR: tetramethylrhodamine
BisTris: 2-[bis(2-hydroxyethyl)amino]-2-(hydroxymethyl)propane-1,3-diol.

## References

[1] Lairson L. L., Henrissat B., Davies G. J., and Withers S. G. (2008) Glycosyltransferases: structures, functions, and mechanisms. Annu. Rev. Biochem. 77, 521–555 18518825 10.1146/annurev.biochem.76.061005.092322

[2] Weadge J. T., and Palcic M. M. (2008) Chemistry of Glycosyltransferases, in Wiley Encyclopedia of Chemical Biology (Begley T. P., ed) Vol. 2, pp. 198–211, Wiley, New York

[3] Yamamoto F., Cid E., Yamamoto M., and Blancher A. (2012) ABO research in the modern era of genomics. Transfus. Med. Rev. 26, 103–118 21945157 10.1016/j.tmrv.2011.08.002

[4] Yamamoto F., Clausen H., White T., Marken J., and Hakomori S. (1990) Molecular genetic basis of the histo-blood group ABO system. Nature 345, 229–233 2333095 10.1038/345229a0

[5] Yamamoto F., and Hakomori S. (1990) Sugar-nucleotide donor specificity of histo-blood group A and B transferases is based on amino acid substitutions. J. Biol. Chem. 265, 19257–19262 2121736

[6] Yamamoto M., Lin X. H., Kominato Y., Hata Y., Noda R., Saitou N., and Yamamoto F. (2001) Murine equivalent of the human histo-blood group ABO gene is a cis-AB gene and encodes a glycosyltransferase with both A and B transferase activity. J. Biol. Chem. 276, 13701–13708 11278752 10.1074/jbc.M010805200

[7] Pesnot T., Jørgensen R., Palcic M. M., and Wagner G. K. (2010) Structural and mechanistic basis for a new mode of glycosyltransferase inhibition. Nat. Chem. Biol. 6, 321–323 20364127 10.1038/nchembio.343 PMC2883747

[8] Rillahan C. D., Antonopoulos A., Lefort C. T., Sonon R., Azadi P., Ley K., Dell A., Haslam S. M., and Paulson J. C. (2012) Global metabolic inhibitors of sialyl- and fucosyltransferases remodel the glycome. Nat. Chem. Biol. 8, 661–668 22683610 10.1038/nchembio.999 PMC3427410

[9] Gloster T. M., Zandberg W. F., Heinonen J. E., Shen D. L., Deng L., and Vocadlo D. J. (2011) Hijacking a biosynthetic pathway yields a glycosyltransferase inhibitor within cells. Nat. Chem. Biol. 7, 174–181 21258330 10.1038/nchembio.520 PMC3202988

[10] Breton C., Fournel-Gigleux S., and Palcic M. M. (2012) Recent structures, evolution and mechanisms of glycosyltransferases. Curr. Opin. Struct. Biol. 22, 540–549 22819665 10.1016/j.sbi.2012.06.007

[11] Evitt A., Tedaldi L. M., and Wagner G. K. (2012) One-step synthesis of novel glycosyltransferase inhibitors. Chem. Commun. (Camb.) 48, 11856–11858 23125983 10.1039/c2cc36798j

[12] Jørgensen R., Pesnot T., Lee H. J., Palcic M. M., and Wagner G. K. (2013) Base-modified donor analogues reveal novel dynamic features of a glycosyltransferase. J. Biol. Chem. 288, 26201–26208 23836908 10.1074/jbc.M113.465963 PMC3764824

[13] Tedaldi L. M., Pierce M., and Wagner G. K. (2012) Optimised chemical synthesis of 5-substituted UDP-sugars and their evaluation as glycosyltransferase inhibitors. Carbohydr. Res. 364, 22–27 23147042 10.1016/j.carres.2012.10.009

[14] Wagstaff B. A., Rejzek M., Pesnot T., Tedaldi L. M., Caputi L., O'Neill E. C., Benini S., Wagner G. K., and Field R. A. (2015) Enzymatic synthesis of nucleobase-modified UDP-sugars: scope and limitations. Carbohydr. Res. 404, 17–25 25662737 10.1016/j.carres.2014.12.005 PMC4340641

[15] Alfaro J. A., Zheng R. B., Persson M., Letts J. A., Polakowski R., Bai Y., Borisova S. N., Seto N. O., Lowary T. L., Palcic M. M., and Evans S. V. (2008) ABO(H) blood group A and B glycosyltransferases recognize substrate via specific conformational changes. J. Biol. Chem. 283, 10097–10108 18192272 10.1074/jbc.M708669200

[16] Persson K., Ly H. D., Dieckelmann M., Wakarchuk W. W., Withers S. G., and Strynadka N. C. (2001) Crystal structure of the retaining galactosyltransferase LgtC from *Neisseria meningitidis* in complex with donor and acceptor sugar analogs. Nat. Struct. Biol. 8, 166–175 11175908 10.1038/84168

[17] Pruitt R. N., Chumbler N. M., Rutherford S. A., Farrow M. A., Friedman D. B., Spiller B., and Lacy D. B. (2012) Structural determinants of *Clostridium difficile* toxin A glucosyltransferase activity. J. Biol. Chem. 287, 8013–8020 22267739 10.1074/jbc.M111.298414 PMC3318759

[18] Ramakrishnan B., Balaji P. V., and Qasba P. K. (2002) Crystal structure of β1,4-galactosyltransferase complex with UDP-Gal reveals an oligosaccharide acceptor binding site. J. Mol. Biol. 318, 491–502 12051854 10.1016/S0022-2836(02)00020-7

[19] Descroix K., Pesnot T., Yoshimura Y., Gehrke S. S., Wakarchuk W., Palcic M. M., and Wagner G. K. (2012) Inhibition of galactosyltransferases by a novel class of donor analogues. J. Med. Chem. 55, 2015–2024 22356319 10.1021/jm201154p

[20] Negishi M., Dong J., Darden T. A., Pedersen L. G., and Pedersen L. C. (2003) Glucosaminylglycan biosynthesis: what we can learn from the x-ray crystal structures of glycosyltransferases GlcAT1 and EXTL2. Biochem. Biophys. Res. Commun. 303, 393–398 12659829 10.1016/s0006-291x(03)00356-5

[21] Pham T. T., Stinson B., Thiyagarajan N., Lizotte-Waniewski M., Brew K., and Acharya K. R. (2014) Structures of complexes of a metal-independent glycosyltransferase GT6 from Bacteroides ovatus with UDP-*N*-acetylgalactosamine (UDP-GalNAc) and its hydrolysis products. J. Biol. Chem. 289, 8041–8050 24459149 10.1074/jbc.M113.545384 PMC3961637

[22] Reinert D. J., Jank T., Aktories K., and Schulz G. E. (2005) Structural basis for the function of *Clostridium difficile* toxin B. J. Mol. Biol. 351, 973–981 16054646 10.1016/j.jmb.2005.06.071

[23] Unligil U. M., Zhou S., Yuwaraj S., Sarkar M., Schachter H., and Rini J. M. (2000) X-ray crystal structure of rabbit *N*-acetylglucosaminyltransferase I: catalytic mechanism and a new protein superfamily. EMBO J. 19, 5269–5280 11032794 10.1093/emboj/19.20.5269 PMC314010

[24] Ramakrishnan B., and Qasba P. K. (2002) Structure-based design of β1,4-galactosyltransferase I (β4Gal-T1) with equally efficient *N*-acetylgalactosaminyltransferase activity: point mutation broadens β4Gal-T1 donor specificity. J. Biol. Chem. 277, 20833–20839 11916963 10.1074/jbc.M111183200

[25] Seto N. O., Palcic M. M., Compston C. A., Li H., Bundle D. R., and Narang S. A. (1997) Sequential interchange of four amino acids from blood group B to blood group A glycosyltransferase boosts catalytic activity and progressively modifies substrate recognition in human recombinant enzymes. J. Biol. Chem. 272, 14133–14138 9162041 10.1074/jbc.272.22.14133

[26] Seto N. O., Palcic M. M., Hindsgaul O., Bundle D. R., and Narang S. A. (1995) Expression of a recombinant human glycosyltransferase from a synthetic gene and its utilization for synthesis of the human blood group B trisaccharide. Eur. J. Biochem. 234, 323–328 8529660 10.1111/j.1432-1033.1995.323_c.x

[27] Laferté S., Chan N. W., Sujino K., Lowary T. L., and Palcic M. M. (2000) Intracellular inhibition of blood group A glycosyltransferase. Eur. J. Biochem. 267, 4840–4849 10903519 10.1046/j.1432-1327.2000.01544.x

[28] Sindhuwinata N., Munoz E., Munoz F. J., Palcic M. M., Peters H., and Peters T. (2010) Binding of an acceptor substrate analog enhances the enzymatic activity of human blood group B galactosyltransferase. Glycobiology 20, 718–723 20154292 10.1093/glycob/cwq019

[29] Kabsch W. (1993) Automatic processing of rotation diffraction data from crystals of initially unknown symmetry and cell constants. J. Appl. Crystallogr. 26, 795–800

[30] McCoy A. J., Grosse-Kunstleve R. W., Storoni L. C., and Read R. J. (2005) Likelihood-enhanced fast translation functions. Acta Crystallogr. D Biol. Crystallogr. 61, 458–464 15805601 10.1107/S0907444905001617

[31] Murshudov G. N., Vagin A. A., and Dodson E. J. (1997) Refinement of macromolecular structures by the maximum-likelihood method. Acta Crystallogr. D Biol. Crystallogr. 53, 240–255 15299926 10.1107/S0907444996012255

[32] Emsley P., and Cowtan K. (2004) Coot: model-building tools for molecular graphics. Acta Crystallogr. D Biol. Crystallogr. 60, 2126–2132 15572765 10.1107/S0907444904019158

[33] Adams P. D., Grosse-Kunstleve R. W., Hung L. W., Ioerger T. R., McCoy A. J., Moriarty N. W., Read R. J., Sacchettini J. C., Sauter N. K., and Terwilliger T. C. (2002) PHENIX: building new software for automated crystallographic structure determination. Acta Crystallogr. D Biol. Crystallogr. 58, 1948–1954 12393927 10.1107/s0907444902016657

[34] Laskowski R. A., MacArthur M. W., Moss D. S., and Thornton J. M. (1993) PROCHECK: a program to check the stereochemical quality of protein structures. J. Appl. Crystallogr. 26, 283–291

[35] Collier A., and Wagner G. K. (2008) A fast synthetic route to GDP-sugars modified at the nucleobase. Chem. Commun. (Camb.) 2, 178–180 18092079 10.1039/b714379f

[36] Pesnot T., Palcic M. M., and Wagner G. K. (2010) A novel fluorescent probe for retaining galactosyltransferases. Chembiochem 11, 1392–1398 20533489 10.1002/cbic.201000013

[37] Pesnot T., and Wagner G. K. (2008) Novel derivatives of UDP-glucose: concise synthesis and fluorescent properties. Org. Biomol. Chem. 6, 2884–2891 18688480 10.1039/b805216f

[38] Wittmann V., and Wong C. H. (1997) ^1^H-Tetrazole as catalyst in phosphomorpholidate coupling reactions: efficient synthesis of GDP-fucose, GDP-mannose, and UDP-galactose. J. Org. Chem. 62, 2144–2147 11671520 10.1021/jo9620066

[39] Soya N., Shoemaker G. K., Palcic M. M., and Klassen J. S. (2009) Comparative study of substrate and product binding to the human ABO(H) blood group glycosyltransferases. Glycobiology 19, 1224–1234 19648353 10.1093/glycob/cwp114

[40] Sheng F., Jia X., Yep A., Preiss J., and Geiger J. H. (2009) The crystal structures of the open and catalytically competent closed conformation of *Escherichia coli* glycogen synthase. J. Biol. Chem. 284, 17796–17807 19244233 10.1074/jbc.M809804200 PMC2719418

[41] Zheng Y., Anderson S., Zhang Y., and Garavito R. M. (2011) The structure of sucrose synthase-1 from *Arabidopsis thaliana* and its functional implications. J. Biol. Chem. 286, 36108–36118 21865170 10.1074/jbc.M111.275974 PMC3195635

[42] Seto N. O., Compston C. A., Evans S. V., Bundle D. R., Narang S. A., and Palcic M. M. (1999) Donor substrate specificity of recombinant human blood group A, B, and hybrid A/B glycosyltransferases expressed in *Escherichia coli*. Eur. J. Biochem. 259, 770–775 10092863 10.1046/j.1432-1327.1999.00086.x

[43] Chen V. B., Arendall W. B. 3rd, Headd J. J., Keedy D. A., Immormino R. M., Kapral G. J., Murray L. W., Richardson J. S., and Richardson D. C. (2010) MolProbity: all-atom structure validation for macromolecular crystallography. Acta Crystallogr. D Biol. Crystallogr. 66, 12–21 20057044 10.1107/S0907444909042073 PMC2803126

[44] Gastinel L. N., Bignon C., Misra A. K., Hindsgaul O., Shaper J. H., and Joziasse D. H. (2001) Bovine alpha1,3-galactosyltransferase catalytic domain structure and its relationship with ABO histo-blood group and glycosphingolipid glycosyltransferases. EMBO J. 20, 638–649 11179209 10.1093/emboj/20.4.638 PMC145412

